# Impact of a multidisciplinary team meeting on patient-reported outcomes at 2 years after lumbar surgery: A prospective comparative exploratory study

**DOI:** 10.1097/MD.0000000000032091

**Published:** 2022-11-25

**Authors:** Sébastien Troussier, Emmanuelle Ferrero, Marie-Martine Lefèvre-Colau, Antoine Feydy, Pierre Guigui, François Rannou, Christelle Nguyen

**Affiliations:** a AP-HP.Centre-Université Paris Cité, Hôpital Cochin, Service de Rééducation et de Réadaptation de l’Appareil Locomoteur et des Pathologies du Rachis, Paris, France; b Université Paris Cité, Faculté DE Santé, UFR DE Médecine, Paris, France; c AP-HP.Centre-Université Paris Cité, Hôpital Européen Georges Pompidou, Service de Chirurgie Orthopédique, Paris, France; d École Nationale Supérieure des Arts et Métiers, Paris, France; e INSERM UMRS-1153, Centre de Recherche Épidémiologie et Statistique, ECaMO Team, Paris, France; f Institut Fédératif de Recherche sur le Handicap, Paris, France; g AP-HP.Centre-Université Paris Cité, Hôpital Cochin, Service de Radiologie, Paris, France; h INSERM UMRS-1124, Toxicité Environnementale, Cibles Thérapeutiques, Signalisation Cellulaire et Biomarqueurs (T3S), Campus Saint-Germain-des-Prés, Paris, France.

**Keywords:** Low back pain, spine surgery, multidisciplinary team meeting

## Abstract

Failed back surgery syndrome is a challenge. We hypothesized that a multidisciplinary team meeting (MTM) may be useful to select patients who are the most likely to benefit from lumbar surgery. We conducted an observational, prospective, comparative, exploratory study. We aimed to compare core clinical patient-reported outcomes at 2 years after lumbar surgery between patients who attended a MTM and those who did not. Patients who underwent lumbar surgery for a degenerative disease, in a single academic orthopedic department, between January and September 2018, were consecutively screened. Eligible patients were surveyed between April and June 2020. Patient-reported outcomes included lumbar and radicular pain, spine-specific activity limitations and health-related quality of life assessed *via* self-administered questionnaires. Outcomes were compared between respondents who attended the MTM and those who did not. Overall, 211 patients underwent lumbar surgery, 108 were eligible and 44 included: 11 attended the MTM and 33 did not. Mean participants’ age was 57.4 (15.4) years, symptom duration was 14.8 (15.3) months, lumbar pain was 51.3 (18.2) and radicular pain was 53.4 (18.6). At 2 years, we found no evidence that lumbar and radicular pain, activity limitations and health-related quality of life differed between the 2 groups. The decrease was −26.8 (41.1) versus −20.8 (30.4) in lumbar pain and −25.5 (43.0) versus −19.5 (27.5) in radicular pain, in participants who attended the MTM versus those who did not, respectively. We found no evidence that core clinical patient-reported outcomes at 2 years after lumbar surgery differed between participants who attended the MTM and those who did not. However, the exploratory design of our study does not allow concluding that MTMs do not have an impact.

## 1. Introduction

Failed back surgery syndrome is a challenge.^[1]^ A reason may be the insufficient detection of patients who present anatomical findings inconsistent with symptoms or who have poor prognostic factors. We hypothesized that a multidisciplinary team meeting (MTM) may be useful to select patients who are the most likely to benefit from lumbar surgery.

According to the United Kingdom National Health Service, MTMs are meetings of a group of professionals, who together make decisions regarding recommended treatments of individual patients.^[2]^ MTMs have become a standard of care.^[3]^ Our MTM was created in the 90s and brings together senior spine specialists in order to streamline services for people for whom a spinal surgery is considered. Studies in bariatric surgery or oncology settings suggest that MTM could influence decision and positively impact outcomes.^[4,5]^ In a US tertiary care center, implementation of a MTM was associated with a decrease in the utilization of lumbar surgery.^[6]^ In the present study, we aimed to compare core clinical patient-reported outcomes,^[7]^ at 2 years after lumbar surgery, between patients who attended the MTM and those who did not.

## 2. Methods

### 2.1. Design

We conducted an observational, prospective, comparative, exploratory study. Outcomes and analyzes were prespecified in the protocol. No changes were made to the methods after the study commencement. Our study is reported in accordance with the strengthening the reporting of observational studies in epidemiology statement.^[8]^

### 2.2. Participants

All patients who underwent spinal surgery, in a single academic orthopedic department, between January 1, 2018, and September 30, 2018, with 1 of the 4 senior surgeons participating in the MTM, were consecutively screened in the computerized database of the department, using International Classification of Diseases-10 codes. Inclusion criteria were: adults ≥ 18 and ≤ 85 years, presenting with a nonspecific lumbar disorder. Exclusion criteria were: other spinal disorders such as cervical arthritis or spondyloarthritis, patients who died within 2 years after surgery, inability to write, read or speak French, and cognitive disorders. Eligible patients were invited by mail, between April and June 2020. Characteristics at the time of surgery and whether the patient did or did not attend the MTM were retrospectively retrieved from medical files.

### 2.3. Interventions

MTM takes place in Spine Rehabilitation Department of Cochin Hospital in Paris. Each Wednesday afternoon, a mean of 15 patients are introduced. They are followed by practitioners from the conventional unit or day care. Final treatment decision leads to perform infiltrative or per os drug therapy and/or physical exercise with physiotherapy and/or spine stay wear or lumbar belt wear and/or surgical therapy for the patient who is suffering from spine disorder. For participants who attend the MTM, decisions are founded on reviews of clinical documentation and diagnostic imaging, after discussion between senior spine specialists (i.e., physicians in physical and rehabilitation medicine, orthopedic surgeons, radiologists and rheumatologists), and involve the patient and possibly his family. For participants who do not attend the MTM, decision to operate is at the discretion of the surgeon. This leads to a least description of the pathology, a least magnetic resonance imaging or computed-tomography scan analysis and a situation where failed back surgery syndrome risk factors are less well spotted. Surgical method is not discussed during the MTM but in a second time in a dedicated meeting only between surgeons from Orthopedic Department of Pompidou Hospital in Paris. Conservative treatments are often sufficient for a majority of patients after the MTM.

### 2.4. Assessments

Participants were prospectively surveyed using self-administered questionnaires for core clinical patient-reported outcomes for nonspecific lumbar pain,^[7]^ at 2 years after lumbar surgery. The primary outcomes were lumbar and radicular pain intensities. They were assessed using a self-administered numerical rating scale (0 [no pain] to 100 [maximum pain]). Secondary outcomes were spine-specific activity limitations and health-related quality of life. Spine-specific activity limitations were assessed using the self-administered Oswestry Disability Index (0 [no limitations] to 100 [maximum limitations]).^[9,10]^ The physical and mental components of health-related quality of life were assessed using the physical component score (9.95 [worst quality of life] to 70.02 [best quality of life]) and the mental component score (5.89 [worst quality of life] to 71.97 [best quality of life]) of the self-administered 12-item short form health survey.^[11,12]^

### 2.5. Statistical analyzes

Statistical analyzes were performed using the SYSTAT13 for Windows^®^ software. Quantitative variables were described by their means and standard deviations, and qualitative variables by their absolute (n/N) and relative (%) frequencies. For comparative analyzes between participants who attended the MTM and those who did not, normally distributed quantitative variables were compared using a Student *t* test, non-normally distributed quantitative variables using a Mann–Whitney test, and frequencies using a Fisher exact test. A *P* value < .05 was statistically significant. We hypothesized a reduction in lumbar pain of 15 on 100 points with a standard deviations of 20 points favoring participants who attended the MTM.^[13]^ With an α risk of 5%, a power of 80% and 1 case for 2 controls, we calculated that 22 participants who attended the MTM and 44 who did not, would be needed.

### 2.6. Ethical consideration

All methods were carried out in accordance with relevant guidelines and regulations. All experimental protocols were approved by our ethics committee (*Comité d’éthique de la recherche AP-HP.5, Assistance-Publique-Hôpitaux de Paris, Hôpitaux Universitaires Paris Centre*, Institutional Review Board registration: #00011928). Informed consent was obtained from all subjects.

## 3. Results

### 3.1. Patients

**Figure 1.**
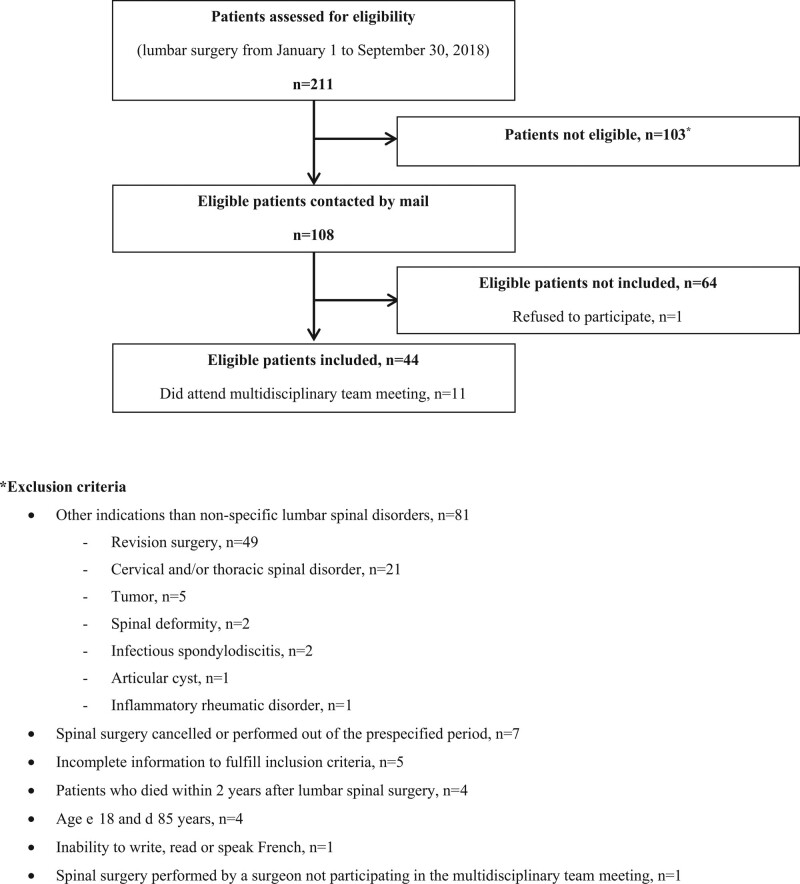
Flow diagram.

Overall, 211 patients underwent lumbar surgery, 108/211 (51%) were eligible and 44/108 (41%) included: 11/44 (25%) attended the MTM and 33/44 (75%) did not (Fig. 1). Mean participants’ age was 57.4 (15.4) years and 7/32 (22%) were on sick leave. Symptom duration was 14.8 (15.3) months, lumbar pain was 51.3 (18.2) and radicular pain was 53.4 (18.6). The percentage of participants on sick leave and symptom duration and lumbar pain were numerically greater in participants who attended the MTM than in those who did not. The percentage of participants having cardiovascular diseases, diabetes, motor and/or sensory deficit, and disc herniation was numerically greater in participants who did not attend the MTM than in those who did (Table 1).

**Table 1:** Characteristics of participants at the time of surgery.

	MTM	No MTM	All
	N = 11	N = 33	N = 44
Age (yrs), mean (SD)	56.7 (17.5)	57.7 (14.8)	57.4 (15.4)
Women, n/N (%)	7/11 (64)	19/33 (58)	26/44 (59)
Body mass index (kg/m^2^), mean (SD)	25.5 (5.6)	27.7 (4.7)^*^	27.1 (4.9)^**^
Higher education, n/N (%)	3/11 (27)	11/33 (33)	14/44 (32)
On sick leave, n/N (%)	4/11 (37)	3/21 (14)	7/32 (22)
**Medical history, n/N (%**)			
Cardiovascular disease	3/11 (27)	15/33 (46)	18/44 (41)
High blood pressure	4/11 (36)	10/33 (30)	14/44 (32)
Depression	3/11 (27)	8/33 (24)	11/44 (25)
Diabetes	1/11 (9)	6/33 (18)	7/44 (16)
**Symptoms**			
Motor and/or sensory deficit in the lower limbs, n/N (%)	1/11 (9)	15/33 (46)	16/44 (27)
Symptom duration, mean (SD)	20.0 (17.4)	13.1 (14.6)	14.8 (15.3)
Lumbar pain (0–100), mean (SD)	62.3 (23.2)	47.6 (16.6)	51.3 (18.2)
Radicular pain (0–100), mean (SD)	52.7 (24.9)	53.6 (16.5)	53.4 (18.6)
**Anatomical findings, n/N (%**)			
Lumbar spinal stenosis	6/11 (55)	18/33 (55)	24/44 (55)
Mixed anatomical findings	4/11(36)	16/33 (49)	20/44 (45)
Disc herniation	4/11 (36)	15/33 (46)	19/44 (43)
Degenerative spondylolisthesis	4/11 (36)	9/33 (27)	13/44 (30)
Modic 1 vertebral endplate changes	2/11 (18)	4/33 (12)	6/44 (14)
Isthmic spondylolisthesis	0/11 (0)	3/33 (9)	3/44 (7)
**Surgical techniques, n/N (%**)			
Laminectomy	7/11 (64)	20/33 (61)	27/44 (61)
Mixed techniques	5/11 (46)	18/33 (55)	23/44 (52)
Discectomy	4/11 (36)	15/33 (46)	19/44 (43)
Posterior lumbar interbody fusion	3/11 (27)	11/33 (33)	14/44 (32)
Anterior lumbar interbody fusion	1/11 (9)	3/33 (9)	4/44 (9)
Transforaminal lumbar interbody fusion	1/11 (9)	2/33 (6)	3/44 (7)
Disc prothesis	0/11 (0)	0/33 (0)	0/44 (0)

MTM = multidisciplinary team meeting; SD = standard deviation.

* n = 28.

** n = 43.

### 3.2. Outcomes

At 2 years, we found no evidence that lumbar and radicular pain, activity limitations and health-related quality of life differed between the 2 groups (Table 2). The decrease was −26.8 (41.1) versus −20.8 (30.4) in lumbar pain and −25.5 (43.0) versus −19.5 (27.5) in radicular pain, in participants who attended the MTM versus those who did not, respectively. Overall, 29/43 (67%) and 26/43 (61%) participants had a pain intensity < 40 of 100 points for lumbar and radicular pain, respectively (Table 3). Except outpatient physiotherapy and strong opioids, all conservative treatments were numerically more frequently self-reported in participants who attended the MTM compared to those who did not (Table 4).

**Table 2:** Primary and secondary outcomes at 2 years after lumbar surgery.

	MTM	No MTM	All	*P* value
	N = 11	N = 33	N = 44	
**Primary outcomes, mean (SD**)				
Lumbar pain (0–100)	35.5 (24.5)	26.7 (26.4)^†^	28.3 (25.9)^∥^	.222^*^
Radicular pain (0–100)	27.3 (27.6)	33.9 (27.6)^†^	32.2 (27.6)^∥^	.449^*^
Δ Lumbar pain (0–100)	−26.8 (41.1)	−20.8 (30.4)^†^	−22.3 (33.1)^∥^	.607^**^
Δ Radicular pain (0–100)	−25.5 (43.0)	−19.5 (27.5)^†^	−21.0 (31.4)^∥^	.599^**^
**Secondary outcomes, mean (SD**)				
ODI (0–100)	20.2 (16.2)	24.9 (19.3)	23.7 (18.5)	.541^*^
SF-12 PCS (9.9–70.0)	47.5 (20.4)	45.0 (14.9)^†^	45.6 (16.3)^¶^	.419^*^
SF-12 MCS (5.9–71.9)	41.5 (6.4)	40.8 (5.2)^†^	41.0 (5.5)^¶^	.754^**^

MCS = mental component score; ODI = Oswestry Disability Index; PCS = physical component score; SD = standard deviation; SF-12 = 12-item short form health survey.

The 2 groups were compared using ^*^the Mann Whithney test and ^**^the Student *t* test. A *P* value < .05 was considered statistically significant.

Δ=variations in scores from time of lumbar surgery to 2 years after.

† n = 32; ^‡^n = 6; ^§^n = 23; ^∥^n = 43; ^¶^n = 29.

**Table 3:** Percentage of patients with pain intensity <40 of 100 points on a numerical rating scale, at 2 years after lumbar spinal surgery.

	MTM	No MTM	Total	*P* value
Lumbar pain < 40 of 100 points, n/N (%)	7/11 (64)	22/32 (69)	29/43 (67)	1.000
Radicular pain < 40 of 100 points, n/N (%)	7/11 (64)	19/32 (59)	26/43 (61)	1.000

MTM = multidisciplinary team meeting.

The 2 groups were compared using the Fisher exact test. A *P* value < .05 was considered statistically significant.

**Table 4:** Treatments received within 2 years after lumbar surgery.

	MTM	No MTM	Total
	N = 11	N = 33	N = 44
**Non-pharmacological treatments, n/N (%**)			
Outpatient physiotherapy	8/11 (73)	19/23 (82)	27/34 (79)
Home-based exercise therapy	8/11 (73)	14/23 (61)	22/34 (65)
Regular physical activity	9/11 (82)	12/32 (38)	21/43 (49)
Lumbar belt	6/11 (55)	9/23 (39)	15/34 (44)
Inpatient multidisciplinary rehabilitation	5/11 (45)	4/23 (17)	9/34 (27)
Osteopathic manipulative treatment	4/11 (36)	5/23 (22)	9/34 (27)
Revision surgery	0/11 (0)	5/33 (15)	5/44 (11)
**Pharmacological treatments, n/N (%**)			
Analgesics			
Nonopioids	10/11 (91)	11/23 (48)	21/34 (62)
Weak opioids^§^	2/11 (18)	6/23(27)	8/34 (24)
Tramadol	3/11 (27)	3/23 (13)	6/34 (18)
Strong opioids^§^	0/11 (0)	3/23 (13)	3/34 (9)
Analgesics for neuropathic pain	3/11 (27)	4/23 (17)	7/34 (21)
Lumbar spinal corticosteroid injection	2/11 (18)	2/23 (9)	4/34 (12)
Topical analgesics	0/11 (0)	2/23 (9)	2/34 (6)

MTM = multidisciplinary team meeting.

§ Weak opioids include codeine and dihydrocodeine; ^§^Strong opioids include morphine, diamorphine, fentanyl, buprenorphine, oxymorphone, oxycodone, and hydromorphone.

## 4. Discussion

In the present study, we found no evidence that core clinical patient-reported outcomes at 2 years after lumbar surgery differed between participants who attended the MTM and those who did not. Several reasons could explain our results.

A first reason could be the lack of difference between the 2 strategies. In order to minimize the impact of clinical heterogeneity on outcomes, we selected participants in the MTM with readily identifiable phenotypes of nonspecific lumbar disorders.^[14,15]^ In these situations, making decisions may not differ with or without MTM. However, the decrease in lumbar and radicular pain was numerically higher in participants who attended the MTM than in those who did not. This positive evolution, despite a greater distribution of poor prognostic factors in participants who attended the MTM than in those who did not,^[16]^ suggests that MTM could be useful in this subgroup of patients, but questionable in others.

A second reason could be our method of recruitment. Patients were not referred at random to the MTM. Indeed, our MTM was designed to discuss complex cases concerning patients for whom conservative treatments have failed. Therefore, participants’ characteristics were unlikely distributed at random between the 2 groups, especially those associated with a poor prognosis.^[16]^ These differences in participants’ characteristics may have impacted outcomes. Adjusted or stratified analyzes should be included in future studies.

A third reason could be the methodological limitations of our study, including its observational design and the recruitment from a single center. We could have minimized these limitations by conducting a multicenter randomized controlled trial. Nevertheless, the random allocation of complex interventions could lead either to an overmedicalization in the simplest cases or insufficient multidisciplinary support in others. Finally, our results could also be explained by our lack of power: we did not achieve our prespecified recruitment and our hypothesis, favoring participants who attended the MTM, was overoptimistic. Further, our sample was too small to conduct adjusted analyzes and to combine effect estimates across groups.

## 5. Conclusion

In summary, we found no evidence that lumbar and radicular pain, activity limitations and health-related quality of life at 2 years differed between participants who attended the MTM and those who did not. However, the exploratory design of our study and its limitations do not allow concluding that MTMs do not have an impact.

## Acknowledgments

For the purchase of stamps, we received a financial assistance by the *Association pour le Développement de l’Enseignement et de la Recherche dans la Pathologie Ostéoarticulaire et Sportive* (ADERPOS). The funding body was neither involved in the design of the study, nor in collection, analysis, and interpretation of data and in writing the manuscript.

## Author contributions

Conception and design of the study: ST, EF, CN. Drafting of the original protocol: ST, EF, CN. Design of the statistical analysis plan: CN. Coordination of the study: CN. Obtaining of funding: FR. Acquisition of data: ST, EF, CN. Drafting of the present manuscript: ST, CN. Reviewing and providing comments on the manuscript: ST, EF, MMLC, AF, PG, FR. Final approval: ST, EF, MMLC, AF, PG, FR, CN.

**Conceptualization:** Sébastien Troussier, Emmanuelle Ferrero, Pierre Guigui, François Rannou, Christelle Nguyen.

**Data curation:** Sébastien Troussier, Emmanuelle Ferrero, Christelle Nguyen.

**Formal analysis:** Sébastien Troussier, Emmanuelle Ferrero, Christelle Nguyen.

**Funding acquisition:** Christelle Nguyen.

**Investigation:** Sébastien Troussier, Emmanuelle Ferrero, Christelle Nguyen.

**Methodology:** Sébastien Troussier, Christelle Nguyen.

**Project administration:** Christelle Nguyen.

**Supervision:** Christelle Nguyen.

**Validation:** François Rannou, Christelle Nguyen.

**Writing – original draft:** Sébastien Troussier, Christelle Nguyen.

**Writing – review & editing:** Sébastien Troussier, Emmanuelle Ferrero, Marie-Martine Lefèvre-Colau, Antoine Feydy, Pierre Guigui, François Rannou, Christelle Nguyen.
